# hCINAP alleviates senescence by regulating MDM2 via p14ARF and the HDAC1/CoREST complex

**DOI:** 10.1093/jmcb/mjad015

**Published:** 2023-03-06

**Authors:** Xinping Huang, Yan Zhao, Min Wei, Ruipeng Zhuge, Xiaofeng Zheng

**Affiliations:** State Key Laboratory of Protein and Plant Gene Research, School of Life Sciences, Peking University, Beijing 100871, China; Department of Biochemistry and Molecular Biology, School of Life Sciences, Peking University, Beijing 100871, China; State Key Laboratory of Protein and Plant Gene Research, School of Life Sciences, Peking University, Beijing 100871, China; Department of Biochemistry and Molecular Biology, School of Life Sciences, Peking University, Beijing 100871, China; State Key Laboratory of Protein and Plant Gene Research, School of Life Sciences, Peking University, Beijing 100871, China; Department of Biochemistry and Molecular Biology, School of Life Sciences, Peking University, Beijing 100871, China; State Key Laboratory of Protein and Plant Gene Research, School of Life Sciences, Peking University, Beijing 100871, China; Department of Biochemistry and Molecular Biology, School of Life Sciences, Peking University, Beijing 100871, China; State Key Laboratory of Protein and Plant Gene Research, School of Life Sciences, Peking University, Beijing 100871, China; Department of Biochemistry and Molecular Biology, School of Life Sciences, Peking University, Beijing 100871, China

**Keywords:** hCINAP, HDAC1/CoREST complex, p14ARF, MDM2, transcription, senescence

## Abstract

Cellular senescence is a major process affected by multiple signals and coordinated by a complex signal response network. Identification of novel regulators of cellular senescence and elucidation of their molecular mechanisms will aid in the discovery of new treatment strategies for aging-related diseases. In the present study, we identified human coilin-interacting nuclear ATPase protein (hCINAP) as a negative regulator of aging. Depletion of cCINAP significantly shortened the lifespan of *Caenorhabditis elegans* and accelerated primary cell aging. Moreover, mCINAP deletion markedly promoted organismal aging and stimulated senescence-associated secretory phenotype in the skeletal muscle and liver from mouse models of radiation-induced senescence. Mechanistically, hCINAP functions through regulating MDM2 status by distinct mechanisms. On the one hand, hCINAP decreases p53 stability by attenuating the interaction between p14ARF and MDM2; on the other hand, hCINAP promotes MDM2 transcription via inhibiting the deacetylation of H3K9ac in the MDM2 promoter by hindering the HDAC1/CoREST complex integrity. Collectively, our data demonstrate that hCINAP is a negative regulator of aging and provide insight into the molecular mechanisms underlying the aging process.

## Introduction

Aging is the process of a continuous loss of tissue and organ function over time (Flatt, 2012). Extensive studies regarding the molecular mechanisms underlying aging may aid in the generation of new therapeutic strategies for aging-related diseases such as cardiovascular disorders, cancer, type 2 diabetes, and neurodegenerative disorders (Campisi et al., 2019). As a major mechanism of aging, cellular senescence has been extensively studied and identified as a pharmaceutical target to improve human health and lifespan (Lopez-Otin et al., 2013). Cellular senescence is triggered by internal and external environmental factors, including DNA damage, oncogene activation, mitochondrial dysfunction, nutrient deprivation, genotoxic agents, and hypoxia (Gorgoulis et al., 2019), and can be categorized into replicative senescence, oncogene-induced senescence, and stress-induced senescence (Takahashi et al., 2018). During the process of senescence, many changes occur at the subcellular and molecular levels, including cell cycle arrest due to high expression of cyclin-dependent kinase inhibitors (e.g. p16 and p21), chromatin consolidation, accumulation of senescence-associated β-galactosidase (SA-β-gal), and emergence of senescence-associated secretory phenotype (SASP) factors (Rodier and Campisi, 2011). The number of senescent cells increases in multiple tissues during aging, leading to the secretion of a range of proinflammatory cytokines that contribute to systemic dysfunction and chronic diseases (Xu et al., 2015); therefore, reducing the expression of p16 or preventing SASP slows the development of senescence. These potential approaches to the therapeutic targeting of senescent cells will be beneficial for overcoming aging-related diseases (Childs et al., 2015).

In recent years, prominent pathways regulating cellular senescence have been comprehensively studied. In proliferating mammalian cells, telomere DNA gradually shortens, causing a DNA damage response. Activated ATM and/or ATR phosphorylate CHK2 and CHK1, which leads to the upregulation of p53 and p21 and subsequent cell cycle arrest in G1/G2 phase (d'Adda di Fagagna, 2008). The *CDKN2a* locus, producing *p16* and *ARF*, is activated in most senescent cells, and the level of p16 expression is increased in tissues during aging (Krishnamurthy et al., 2004). During Ras mutation-induced DNA damage, the expression of p21 is promoted through the Raf/MeF and mouse double minute 2 (MDM2)/p53 pathways, which eventually leads to the occurrence of cell senescence (Takaoka et al., 2004). A recent study reported that MDM2 functions by suppressing p53-mediated apoptosis, and MDM2 transcription is also changed in senescent fibroblasts (Sturmlechner et al., 2022). Considering the complex senescence regulatory network, it is necessary to identify new regulators of senescence in order to better understand the underlying mechanisms of aging.

Human coilin-interacting nuclear ATPase protein (hCINAP), also known as adenylate kinase 6, is highly conserved in eukaryotes (Ren et al., 2005; Santama et al., 2005). Depletion of hCINAP in *Caenorhabditis elegans* and *Arabidopsis* markedly inhibits worm growth (Zhai et al., 2006) and plant bolting (Feng et al., 2012), respectively. In human cells, hCINAP has been demonstrated to play key roles in many biological processes and regulate tumorigenesis by distinct mechanisms (Zhang et al., 2010, 2014; Bai et al., 2016; Ji et al., 2017); however, the physiological function of hCINAP in aging has not yet been elucidated.

In the present study, we demonstrate that hCINAP prevents cellular senescence by controlling MDM2 status. hCINAP depletion aggravates aging phenotypes not only through the regulation of the p14ARF–MDM2–p53 pathway but also through the downregulation of MDM2 transcription by promoting the assembly of the histone deacetylase 1/corepressor for element 1-silencing transcription factor (HDAC1/CoREST) transcription repression complex. Our data suggest that hCINAP is a potent target for delaying aging and ameliorating aging-related disorders.

## Results

### Loss of cCINAP (hCINAP homologous protein) reduces the lifespan of *C. elegans*

**Figure 1:**
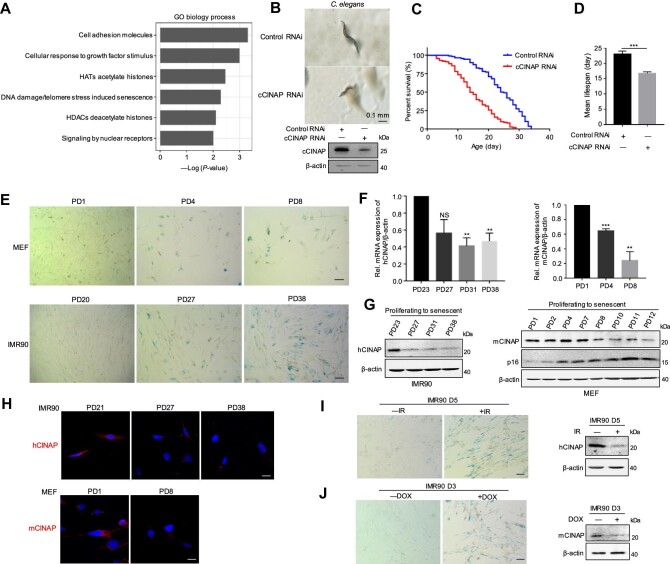
hCINAP levels decrease during cellular senescence. (**A**) Enriched biological pathway analysis of *hCINAP^WT^* and *hCINAP^–/–^* HEK293T cells. Significant Gene Ontology (GO) terms and their enrichment scores are displayed. (**B**) Knockdown of cCINAP in *C. elegans* by RNA interference (RNAi). *C. elegans* were cultured at 20°C. Images show the morphological sizes of control and cCINAP RNAi worms under a stereomicroscope at 10 × 2.5 magnification. Scale bar, 0.1 mm. Knockdown of cCINAP was confirmed by immunoblotting. (**C**) Survival curves for control and cCINAP RNAi *C. elegans*. The numbers of surviving and dead *C. elegans* were counted daily. (**D**) The average lifespan of control and cCINAP RNAi *C. elegans*. Data are expressed as mean ± SEM (*n* = 3). Student's *t*-test, ***P < 0.001. (**E**) SA-β-gal levels in MEFs and IMR90 cells undergoing replicative senescence. Scale bar, 50 μm. (**F**) mRNA expression levels of hCINAP in different passages of IMR90 cells and MEFs were assessed by RT-qPCR. Data are expressed as mean ± SEM (*n* = 3). Student's *t*-test, **P < 0.01, ***P < 0.001. NS, not significant. (**G**) Protein expression levels of hCINAP and p16 during senescence in IMR90 cells and MEFs were examined by immunoblotting using the indicated antibodies. β-actin served as a loading control. (**H**) Immunofluorescence staining of hCINAP in different passages of IMR90 cells and MEFs. Scale bar, 10 μm. (**I** and **J**) IMR90 cells (PD21) were rendered senescent by either exposure to 10 Gy IR followed by culture for 5 days or treatment with DOX (2 μg/ml) for 24 h followed by culture for 3 days. Cellular senescence was detected by SA-β-gal staining. Scale bar, 50 μm (left). hCINAP protein levels were assessed by immunoblotting (right).

To explore the physiological significance of hCINAP, total RNA was extracted from *hCINAP*^–/–^ cells and subjected to RNA-sequencing. Enriched biological pathway analysis revealed that hCINAP is involved in aging (Figure 1A). Moreover, to determine the role of hCINAP in senescence, cCINAP (hCINAP homologous protein) was knocked down, and the effects of cCINAP depletion were investigated in *C. elegans*. In comparison with the control group, cCINAP-depleted *C. elegans* displayed a smaller body size (Figure 1B). More importantly, knockdown of cCINAP significantly decreased the lifespan of *C. elegans* (Figure 1C and D). These results demonstrate that cCINAP suppresses senescence in *C. elegans*.

### hCINAP levels decrease during cellular senescence

Mouse embryonic fibroblasts (MEFs) and IMR90 cells, which display senescence-associated phenotypes, were used to examine senescence-associated changes in hCINAP at the cellular level. MEFs isolated from mice and IMR90 cells were continuously cultured to construct replicative cellular senescence models. As expected, SA-β-gal activity in MEFs and IMR90 cells increased gradually with passage (Figure 1E), indicating replicative senescence. To clarify whether the expression levels of hCINAP change during cellular senescence, total RNA was extracted from different passages of MEFs and IMR90 cells and subjected to real-time quantitative polymerase chain reaction (RT-qPCR). The results indicated that the mRNA levels of hCINAP were reduced during cellular senescence (Figure 1F). In accordance with this, the protein levels of hCINAP, as assessed by immunoblotting and immunofluorescence, were markedly decreased in senescent MEFs and IMR90 cells (Figure 1G and H).

Subsequently, the change in hCINAP expression in cells displaying stress-induced senescence was examined. IMR90 cells were exposed to ionizing radiation (IR) or treated with doxorubicin (DOX), which represents the universal DNA damage-induced model of senescence. SA-β-gal staining demonstrated that IR or DOX treatment indeed accelerated cellular senescence, and the levels of hCINAP were decreased (Figure 1I and J). Collectively, these results indicate that the expression of hCINAP decreases during cellular senescence.

### Loss of hCINAP promotes cellular senescence

**Figure 2:**
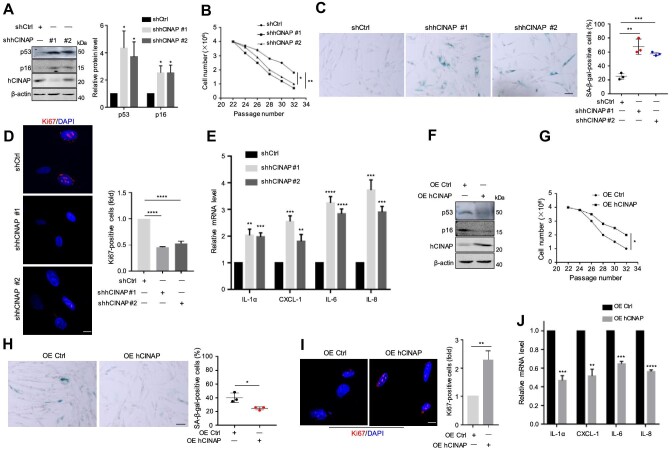
Depletion of hCINAP promotes cellular senescence. (**A**) hCINAP knockdown increased the abundance of the aging markers, p53 and p16, in IMR90 cells (left). Quantification results are shown (right). (**B**) hCINAP depletion inhibited IMR90 cell growth. (**C**) hCINAP knockdown resulted in stronger SA-β-gal staining in IMR90 cells. Scale bar, 50 μm. One-way ANOVA with Dunnett's multiple comparison was performed (right panel). (**D**) Immunofluorescence staining of Ki67 in control (shCtrl) and hCINAP-depleted (shhCINAP) IMR90 cells. Scale bar, 10 μm. Quantification of Ki67-positive cells was performed using the ImageJ software. (**E**) mRNA levels of the SASP factors IL-1α, CXCL-1, IL-6, and IL-8 in control and hCINAP-depleted IMR90 cells were assessed by RT-qPCR. (**F**) Protein expression levels of p53 and p16 in control (OE Ctrl) and hCINAP-overexpressing (OE hCINAP) IMR90 cells were measured by immunoblotting. (**G**) hCINAP overexpression promoted the growth of IMR90 cells. (**H**) hCINAP overexpression reduced the number of SA-β-gal-positive IMR90 cells. Scale bar, 50 μm. (**I**) Immunofluorescence staining of Ki67 and quantification of Ki67-positive cells in control and hCINAP-overexpressing IMR90 cells. Scale bar, 10 μm. (**J**) Effects of hCINAP overexpression on the mRNA levels of SASP factors in IMR90 cells were analyzed by RT-qPCR. Quantitative results are expressed as mean ± SEM (*n* = 3). Student's *t*-test, *P < 0.05, **P < 0.01, ***P < 0.001, ****P < 0.0001.

To further investigate the role of hCINAP in cellular senescence, hCINAP was stably knocked down in IMR90 cells using two different hCINAP shRNA lentiviruses (shhCINAP #1 and shhCINAP #2), and the effects of hCINAP depletion on p53 and p16, biomarkers of aging, were detected by immunoblotting. In comparison with control IMR90 cells, the protein levels of p53 and p16 were markedly increased in hCINAP-depleted cells (Figure 2A), suggesting that depletion of hCINAP leads to premature senescence. Moreover, the cell division rate of hCINAP-depleted cells was significantly reduced in comparison with that of control cells, with most hCINAP-depleted cells entering a senescent state (Figure 2B). Consistent with these observations, knockdown of hCINAP increased SA-β-gal activity in IMR90 cells (Figure 2C), and Ki67 staining confirmed that hCINAP depletion decreased replicative activity (Figure 2D). To verify whether hCINAP knockdown promotes cellular senescence, RT-qPCR was performed to detect the expression levels of SASP factors, including IL-1α, CXCL-1, IL-6, and IL-8, in hCINAP-depleted IMR90 cells. The results showed that hCINAP knockdown led to increased mRNA levels of SASP factors (Figure 2E), suggesting that depletion of hCINAP causes cellular senescence.

In addition, hCINAP was overexpressed in IMR90 cells to explore whether the senescent phenotype was alleviated. The results showed that overexpression of hCINAP led to reduced abundances of p53 and p16 proteins (Figure 2F), and the cell division rate of hCINAP-overexpressing cells was also significantly increased in comparison with that of control cells (Figure 2G). Moreover, overexpression of hCINAP delayed cellular senescence, as indicated by significantly decreased SA-β-gal staining (Figure 2H), increased proliferation rate (Figure 2I), and decreased expression of SASP factors (Figure 2J). Taken together, these data indicate that hCINAP is a negative regulator of senescence.

### hCINAP attenuates the interaction between p14ARF and MDM2

To elucidate the molecular mechanism underlying the cellular senescence phenotypes caused by hCINAP deficiency, hCINAP-interacting proteins were investigated using immunoprecipitation (IP) followed by mass spectrometry. Among the hCINAP-associated proteins, p14ARF was identified (Figure 3A), which plays a crucial role in cellular senescence by regulating cell cycle arrest and/or apoptosis in both p53-dependent and p53-independent manners (Ozenne et al., 2010). Co-immunoprecipitation (co-IP) showed that hCINAP was associated with p14ARF (Figure 3B; Supplementary Figure S1). Furthermore, a direct interaction between hCINAP and p14ARF was confirmed by *in vitro* His-pulldown experiments (Figure 3C), and Duolink® proximity ligation assay (PLA) indicated the *in situ* binding between hCINAP and p14ARF (Figure 3D). These data demonstrate that p14ARF is a binding partner of hCINAP.

**Figure 3:**
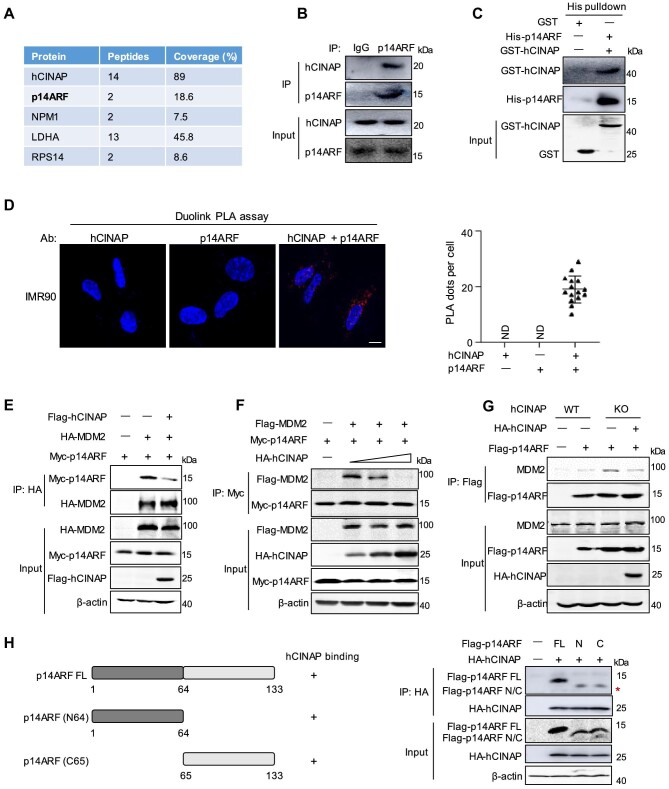
hCINAP attenuates the interaction between p14ARF and MDM2. (**A**) Interactors of hCINAP were identified by IP–mass spectrometry using HEK293T cells transfected with Flag-hCINAP. Major hits are shown. (**B**) Co-IP assays were performed using the indicated antibodies to detect the association between endogenous hCINAP and p14ARF. (**C**) The direct interaction between hCINAP and p14ARF was confirmed by *in vitro* pulldown using His-p14ARF and GST-hCINAP proteins purified from *Escherichia coli*. (**D**) The *in situ* interaction between hCINAP and p14ARF in IMR90 cells was assessed by Duolink® PLA using anti-hCINAP and anti-p14ARF antibodies. Scale bar, 10 μm. Quantitative data from the PLA dots indicating a hCINAP–p14ARF interaction are expressed as mean ± SEM (right). ND, not detectable. (**E** and **F**) The effect of

**Figure 3:**
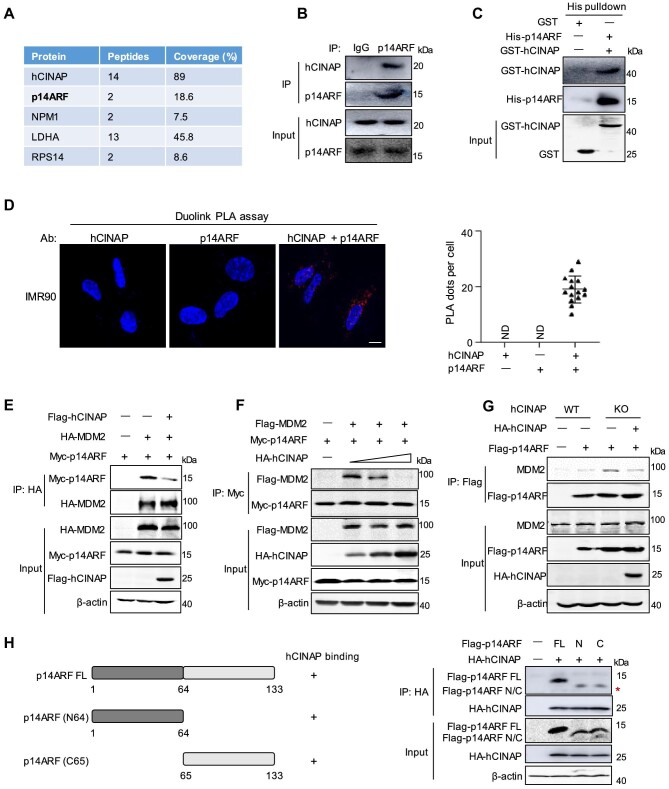
*(Continued)* hCINAP on the interaction between p14ARF and MDM2 was examined by co-IP using HEK293T cells transfected with the indicated plasmids. (**G**) Depletion of hCINAP enhanced the p14ARF–MDM2 interaction. Wild-type (WT) HEK293T cells, hCINAP-knockout (KO) HEK293T cells, and hCINAP-knockout HEK293T cells transfected with HA-hCINAP were subjected to IP analysis. (**H**) Schematic representation of full-length (FL), N-terminus truncation (N64), and C-terminus truncation (C65) of p14ARF (left). HEK293T cells transfected with the indicated plasmids were subjected to co-IP assays to examine the interactions between hCINAP and p14ARF truncations (right).

It is well known that p14ARF interacts with MDM2, an E3-ubiquitin ligase of p53, inducing MDM2 nucleolar localization and inactivation, which prevents MDM2-modulated p53 ubiquitination and degradation (Weber et al., 1999). Since hCINAP interacts with p14ARF, the effect of hCINAP on the interaction between p14ARF and MDM2 was investigated by co-IP. Overexpression of hCINAP attenuated the p14ARF–MDM2 interaction (Figure 3E) in a dose-dependent manner (Figure 3F). Furthermore, *hCINAP*-knockout HEK293T cells were created using the CRISPR–Cas9 system, and the effect of hCINAP depletion on the p14ARF–MDM2 interaction was evaluated. In comparison with wild-type cells, knockout of hCINAP significantly promoted the interaction between p14ARF and MDM2, and overexpression of Flag-hCINAP in *hCINAP*-knockout cells attenuated this interaction (Figure 3G). It has been reported that the N-terminal domain of p14ARF interacts with MDM2 (Zhang et al., 1998). Here, we found that both the N-terminal domain and the C-terminal region of p14ARF were responsible for its interaction with hCINAP (Figure 3H). These results indicate that hCINAP inhibits the p14ARF–MDM2 interaction by competitively binding to p14ARF.

### hCINAP decreases p53 stability via the p14ARF–MDM2–p53 pathway

**Figure 4:**
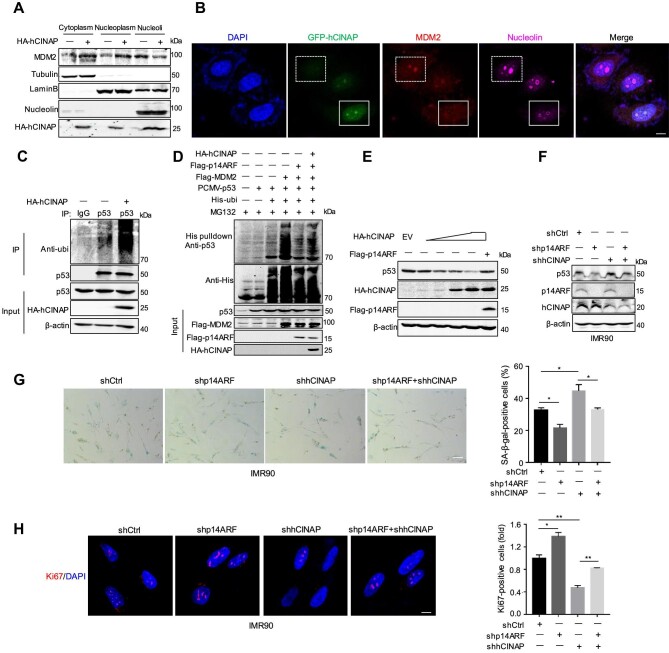
hCINAP decreases p53 stability via the p14ARF–MDM2–p53 pathway. (**A**) The influence of hCINAP on the subcellular localization of MDM2 was examined by subcellular fractionation using HEK293T cells transfected with the indicated plasmids. Tubulin, Lamin B, and nucleolin served as markers for the cytoplasm, nucleoplasm, and nucleoli, respectively. (**B**) HeLa cells transfected with GFP-hCINAP were immunostained using anti-MDM2 (red) and anti-nucleolin (wine red) antibodies. Scale bar, 10 μm. (**C**) hCINAP promoted the ubiquitination of p53. HCT116 cells transfected with the indicated plasmids were subjected to denatured IP assays. (**D**) The p53 ubiquitination levels in HEK293T cells transfected with the indicated plasmids were analyzed by His-ubiquitin pulldown. (**E**) HCT116 cells transfected with varying concentrations of hCINAP and/or p14ARF were subjected to immunoblotting using the indicated antibodies to examine the effect of hCINAP on p53 expression in the presence or absence of p14ARF. (**F**) hCINAP knockdown promoted p53 abundance in IMR90 cells, as demonstrated by immunoblotting using the indicated antibodies. (**G**) SA-β-gal staining of control (shCtrl) and p14ARF-knockdown (shp14ARF) and/or hCINAP-knockdown (shhCINAP) IMR90 cells. Scale bar, 50 μm. (**H**) Immunofluorescence staining of Ki67 and quantification of Ki67-positive cells in shCtrl and shp14ARF and/or shhCINAP IMR90 cells. Scale bar, 10 μm. Quantitative results in **G** and **H** are expressed as mean ± SEM (*n* = 3). Student's *t*-test, *P < 0.05, **P < 0.01.

Since previous studies suggest that p14ARF prevents MDM2 from interacting with p53 in the cytoplasm and nucleus by sequestering MDM2 in the nucleolus (Ko et al., 2016), nucleolar isolation experiments were performed to detect the effects of hCINAP on the subcellular localization of p14ARF and MDM2. The abundance of MDM2 increased in the nucleoplasm but decreased in the nucleolus in cells overexpressing hCINAP (Figure 4A). Consistently, overexpression of GFP-hCINAP led to the release of MDM2 from the nucleolus into the nucleoplasm (Figure 4B). These results demonstrate that hCINAP promotes the release of MDM2 from the nucleolus by attenuating the p14ARF–MDM2 interaction.

Subsequently, His-ubiquitin pulldown assays and denatured IP assays were conducted to determine whether hCINAP promotes the ubiquitination of p53 and prevents p53 stabilization by freeing MDM2 to bind to p53. hCINAP notably promoted the ubiquitination of p53 (Figure 4C; Supplementary Figure S2), while overexpression of p14ARF inhibited MDM2-mediated p53 ubiquitination, which was efficiently rescued by co-expression of hCINAP (Figure 4D). Consistently, the protein level of p53 was decreased by hCINAP in a dose-dependent manner, which was recovered by p14ARF overexpression (Figure 4E). These results demonstrate that hCINAP increases MDM2-mediated p53 ubiquitination by attenuating the interaction between p14ARF and MDM2.

Since knockdown of hCINAP accelerated cellular senescence (Figure 2) and hCINAP decreased p53 stability by binding to p14ARF and reducing the p14ARF–MDM2 interaction (Figure 4A–E), we sought to determine whether hCINAP regulation of cellular senescence is dependent on p14ARF. IMR90 cells stably expressing p14ARF shRNA and/or hCINAP shRNA were generated, and the expression levels of p53 were evaluated with a view to characterize the senescent state. The protein level of p53 was substantially decreased in p14ARF-depleted cells and increased in hCINAP-depleted cells, whereas hCINAP depletion no longer promoted p53 protein level in p14ARF-depleted cells (Figure 4F). Moreover, a similar pattern of SA-β-gal-positive cells was observed, i.e. knockdown of p14ARF significantly reduced and knockdown of hCINAP increased the number of SA-β-gal-positive cells, whereas knockdown of hCINAP in p14ARF-depleted cells resulted in SA-β-gal staining similar to that displayed in wild-type cells (Figure 4G). Consistently, Ki67 staining showed that the replicative activity of cells was increased by p14ARF knockdown but decreased by hCINAP knockdown, while knockdown of hCINAP in p14ARF-depleted cells only slightly reduced replicative activity (Figure 4H). Because p53 is a central player in regulating cellular senescence (Wu and Prives, 2018), we also investigated whether hCINAP-mediated cellular senescence is dependent on p53. As expected, p53 depletion substantially decreased and hCINAP knockdown increased the protein levels of p21 and p16 in IMR90 cells, whereas hCINAP knockdown no longer promoted the protein levels of p21 and p16 in p53-depleted IMR90 cells (Supplementary Figure S3A). In accordance with this observation, knockdown of p53 significantly reduced the number of SA-β-gal-positive cells, whereas hCINAP knockdown only slightly increased the number of SA-β-gal-positive cells in p53-depleted cells (Supplementary Figure S3B). These data indicate that hCINAP functions as an upstream regulator of cellular senescence in a p14ARF/p53-dependent manner by modulating the p14ARF–MDM2–p53 axis.

### hCINAP promotes MDM2 transcription via inhibition of H3K9ac deacetylation in the MDM2 promoter by blocking the assembly of the HDAC1/CoREST complex

**Figure 5:**
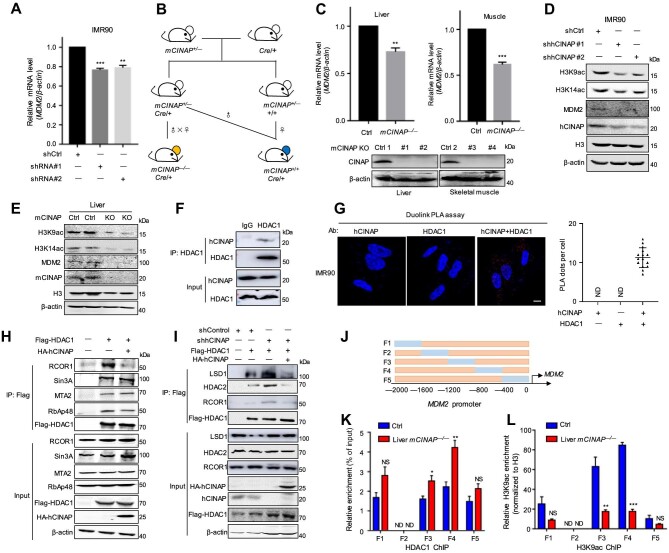
hCINAP positively regulates *MDM2* transcription via inhibiting the deacetylation of H3K9ac in the *MDM2* promoter by blocking HDAC1/CoREST complex assembly. (**A**) MDM2 mRNA levels in control and hCINAP-depleted IMR90 cells were assessed by RT-qPCR. (**B**) Schematic of the experimental procedure for the generation of tissue-specific mCINAP-knockout mouse model. *mCINAP^flp/+^* mice were crossed with tissue (skeletal muscle or liver)-specific *Cre*/+ mice to generate *mCINAP^+/–^;Cre/+* and *mCINAP*^+/–^;*mCINAP^+/+^* mice. Subsequently, *mCINAP*^+/–^;*Cre*/+ mice (male) were crossed with *mCINAP*^+/–^;*Cre*/+ mice (female) to generate *mCINAP*^–/–^;*Cre*/+ mice (*mCINAP*^–/–^). *mCINAP*^+/–^;*Cre*/+ mice were crossed with *mCINAP*^+/–^;*mCINAP^+/+^* mice to generate *mCINAP*^+/+^;*Cre*/+ mice (Ctrl). (**C**) MDM2 mRNA levels in liver/muscle tissues from control and liver/skeletal muscle-specific *mCINAP*^–/–^ mice were assess by RT-qPCR. β-actin was used as the loading control. (**D** and **E**) IMR90 extracts (**D**) and liver tissues (**E**) were subjected to immunoblotting for H3K9/14ac and MDM2 protein levels. β-actin and H3 were used as the loading controls. (**F**) The interaction between hCINAP and HDAC1 was confirmed by co-IP in HEK293T cells. (**G**) The interaction between hCINAP and HDAC1 was detected in IMR90 cells by PLA. Scale bar, 10 μm. Quantitative data from the PLA dots indicating a hCINAP–HDAC1 interaction are expressed as mean ± SEM. (**H**) The effect of hCINAP on the interactions between HDAC1 and four major components (SIN3A, MTA2, RCOR1, and RbAp48) of the complexes was examined by co-IP using HEK293T cells transfected with the indicated plasmids. (**I**) Depletion of hCINAP enhanced the interaction of HDAC1 with the three major components (LSD1, HDAC2, and RCOR1) of the CoREST complex. HEK293T cells transfected with the indicated shRNAs and plasmids were subjected to IP analysis. (**J**) A model of truncated primers for different regions of the *MDM2* promoter. (**K** and **L**) ChIP assays were performed using the chromatin prepared from liver tissues of control and liver-specific *mCINAP*^–/–^ mice. The chromatin was immunoprecipitated using rabbit IgG or an antibody against HDAC1 (**K**) or H3K9ac (**L**), and precipitated genomic DNA was analyzed by RT-qPCR using different primers for different regions of the *MDM2* promoter. ND, not detectable. Quantitative results are expressed as mean ± SEM (*n* = 3). Student's *t*-test, *P < 0.05, **P < 0.01, ***P < 0.001. NS, not significant.

MDM2 controls the ability of p53 to regulate the transcription of numerous genes involved in DNA repair, cell cycle arrest, and senescence. MDM2 dysregulation results in p53-mediated aging phenotypes (Chibaya et al., 2021) and *MDM2* transcriptional changes during the senescence process in human fibroblasts (Sturmlechner et al., 2022); therefore, we sought to investigate whether *MDM2* transcription is also regulated by hCINAP during aging. hCINAP was knocked down in IMR90 cells to induce senescence, and the expression of *MDM2* was analyzed. Indeed, hCINAP depletion led to the downregulation of MDM2 mRNA levels (Figure 5A). To further confirm the regulatory role of hCINAP in maintaining *MDM2* transcription *in vivo*, a skeletal muscle/liver-specific mCINAP-knockout mouse model was constructed (Figure 5B), since deletion of mCINAP (hCINAP homolog) in mice results in embryonic lethality (Bai et al., 2016). The liver/muscle tissues were collected from control and liver/skeletal muscle-specific *mCINAP*^–/–^ mice, respectively, and the MDM2 mRNA levels were significantly lower in the tissues from liver/skeletal muscle-specific *mCINAP*^–/–^ mice (Figure 5C).

Next, to clarify the manner in which hCINAP regulates *MDM2* transcription, we investigated the effect of hCINAP knockdown on the acetylation status of H3K9 and H3K14, which has been demonstrated to play a key role in transcriptional regulation. Decreases in H3K9/14ac levels were observed in hCINAP-depleted IMR90 cells and liver tissues from liver-specific *mCINAP*^–/–^ mice (Figure 5D and E), suggesting that hCINAP may regulate *MDM2* transcription by modulating histone acetylation. To elucidate the underlying mechanism, a small-scale screening was conducted, which revealed that hCINAP inhibited HDAC1-catalyzed deacetylation of H3K9ac (Supplementary Figure S4A). Co-IP and PLA further demonstrated that hCINAP interacted with HDAC1 (Figure 5F and G; Supplementary Figure S4B) but not HDAC2 (Supplementary Figure S4C). HDAC1 is a key component of four distinct transcription repression complexes, i.e. Sin3, nucleosome remodeling and deacetylation (NuRD), CoREST, and NCoR/SMRT complexes (Hayakawa and Nakayama, 2011), and exerts deacetylating activity through these complexes, mainly of H3K4 and H3K9 (Roopra et al., 2004; Shi et al., 2005). Therefore, the influence of hCINAP on the interactions between HDAC1 and the four major components, SIN3A, MTA2, RCOR1, and RbAp48, of these complexes was examined. Through the interaction with HDAC1, hCINAP attenuated HDAC1–RCOR1 binding to block the assembly of the CoREST complex (Figure 5H). Furthermore, overexpression of hCINAP inhibited the interaction between HDAC1 and the major components of the CoREST complex, including LSD1, HDAC2, and RCOR1 (Supplementary Figure S4D). Knockdown of hCINAP substantially promoted the interaction between HDAC1 and components of the CoREST complex, and overexpression of hCINAP in *hCINAP*-knockdown cells attenuated these interactions (Figure 5I). These results suggest that hCINAP inhibits HDAC1/CoREST complex assembly by competitively binding to HDAC1.

Since hCINAP appeared to regulate the integrity of the HDAC1/CoREST complex, we speculated that hCINAP may regulate *MDM2* transcription by affecting the binding of HDAC1/CoREST and acetylated histones to the *MDM2* promoter. Chromatin immunoprecipitation (ChIP)–qPCR was performed to assess the binding of HDAC1 and H3K9ac to different *MDM2* promoter regions (Figure 5J) using samples extracted from liver tissues of control and liver-specific *mCINAP*^–/–^ mice. The results showed that HDAC1 bound to the –800 to –400 region of the *MDM2* promoter, which was significantly increased following mCINAP depletion (Figure 5K). Consistently, mCINAP depletion led to a marked downregulation of H3K9ac levels in the –800 to –400 region of the *MDM2* promoter (Figure 5L). These results indicate that hCINAP positively regulates *MDM2* transcription by blocking the assembly of the HDAC1/CoREST complex and subsequently inhibiting deacetylation of H3K9ac in the *MDM2* promoter.

### mCINAP depletion aggravates senescence-associated phenotypes *in vivo*

The aforementioned results indicate that hCINAP is essential for delaying cellular senescence, and its expression level is associated with the process of senescence. Given that cellular senescence may accelerate aging following the exhaustion of tissue regenerative capacity (Campisi, 2013) and the MDM2/p53 pathway participates in cellular senescence during tissue development, we next sought to investigate whether hCINAP–MDM2–p53 signaling affects aging *in vivo*. A therapy-challenged mouse model was used to examine the effects of hCINAP on senescent cells and organismal aging. Cellular senescence was induced by the exposure of skeletal muscle/liver-specific *mCINAP^–/–^* mice to whole-body irradiation at a sublethal dose (5 Gy), and tissue samples (skeletal muscle and liver) were collected at different ages to detect senescence-associated phenotypes (Figure 6A). Of note, all mice that underwent irradiation developed an abnormal body appearance, such as grey hair, which was markedly aggravated by mCINAP depletion in the skeletal muscle/liver (Figure 6B).

**Figure 6:**
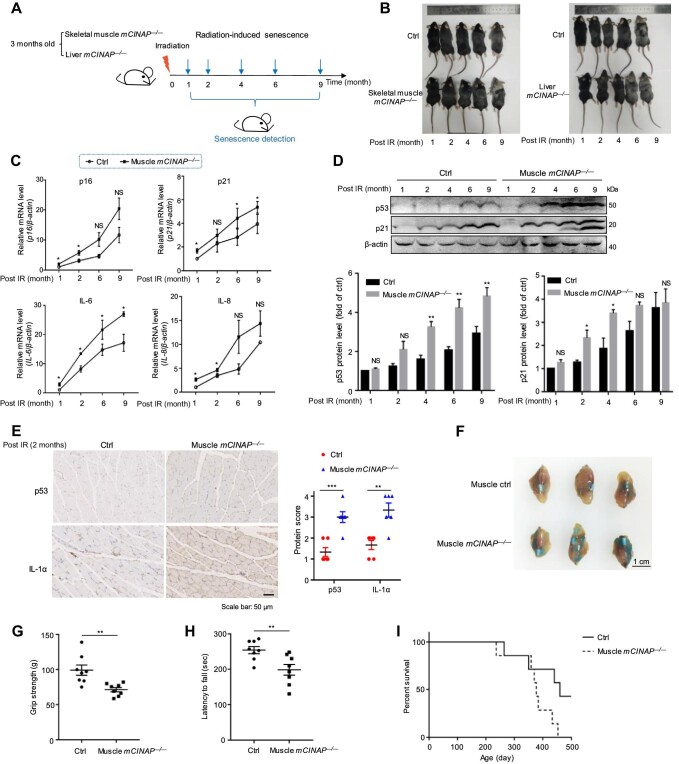
mCINAP depletion aggravates senescence-associated phenotypes *in vivo*. (**A**) Schematic of the experimental procedure for mice subjected to whole-body irradiation and physical function tests. (**B**) Whole-body snapshot comparison of skeletal muscle/liver-specific *mCINAP*^–/–^ and control mice at different ages following exposure to irradiation. (**C**) The mRNA expression levels of senescence markers (p16 and p21) and key SASP factors (IL-6 and IL-8) in the skeletal muscle from control and skeletal muscle-specific *mCINAP*^–/–^ mice. (**D**) Protein expression levels of senescence markers (p16 and p21) in the skeletal muscle from control and skeletal muscle-specific *mCINAP*^–/–^ mice. (**E**) Immunohistochemistry of p53 and IL-1α expression in the skeletal muscle from control and skeletal muscle-specific *mCINAP*^–/–^ mice. Scale bar, 50 μm. Images were analyzed using the ImageJ software. (**F**) Representative images of SA-β-gal staining in skeletal muscle tissues from control and skeletal

**Figure 6:**
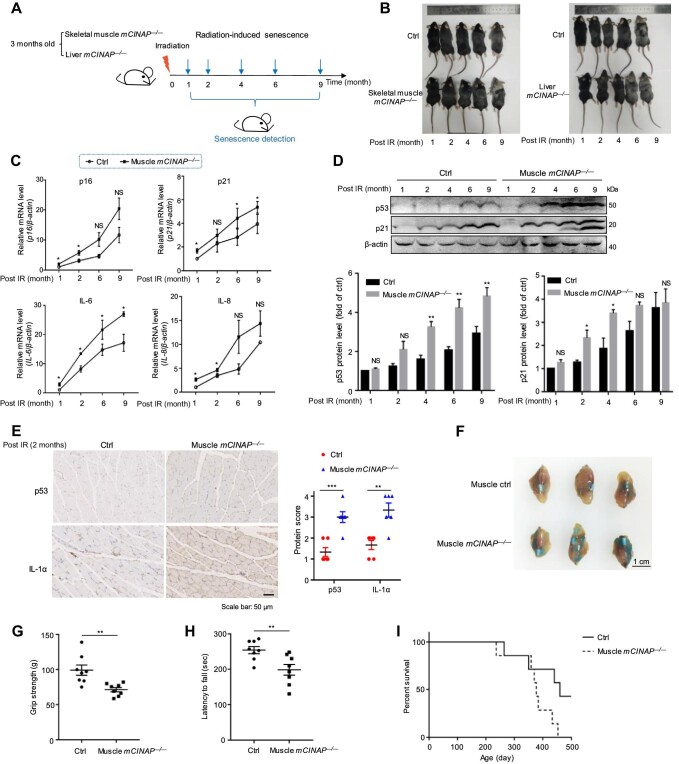
*(Continued)* muscle-specific *mCINAP*^–/–^ male mice (*n* = 3). Scale bar, 1 cm. (**G**) The grip strength test of control and skeletal muscle-specific *mCINAP*^–/–^ mice (12-month-old, male, *n* = 8). (**H**) The motor performance test of mice as in **G**. The staying time was recorded from the mouse starting rotation of the rotarod model to falling on the switch. (**I**) Kaplan–Meier survival curves for IR-challenged control and skeletal muscle-specific *mCINAP*^–/–^ mice (male, *n* = 7). Quantitative results in **C, D, E, G**, and **H** are expressed as mean ± SEM (*n* = 3). Student's *t*-test, *P < 0.1, **P < 0.01, ***P < 0.001. NS, not significant.

To assess the effects of mCINAP depletion on the expression levels of senescence markers and key SASP factors, total RNA and protein were extracted from skeletal muscle and liver tissues of control and skeletal muscle/liver-specific *mCINAP^–/–^* mice at different ages and subjected to RT-qPCR and immunoblotting, respectively. The mRNA levels of p16, p21, IL-6, and IL-8 increased in the skeletal muscle or liver as mice aged. Moreover, the mRNA levels of these senescence markers and SASP factors were higher in mCINAP-depleted skeletal muscle/liver tissues than in control tissues (Figure 6C; Supplementary Figure S5A). The protein levels of the senescence markers p53 and p21 changed in the same pattern (Figure 6D; Supplementary Figure S5B). Furthermore, immunohistochemistry revealed stronger p53 and IL-1α staining in the muscle and liver of skeletal muscle/liver-specific *mCINAP*^–/–^ mice compared with that in control mice (Figure 6E; Supplementary Figure S5C). In addition, we observed intenser SA-β-gal staining in skeletal muscle and liver tissues from skeletal muscle/liver-specific *mCINAP*^–/–^ mice compared with control mice, suggesting that knockout of mCINAP resulted in a higher degree of cellular senescence in skeletal muscle and liver tissues (Figure 6F; Supplementary Figure S5D). Given that depletion of mCINAP in tissues has been detected to aggravate various aging phenotypes, we further tested whether mCINAP depletion also impairs the behavioral phenotypes and lifespan of mice. Relative to the control mice, decreased grip strength and reduced motor skills were observed in skeletal muscle-specific *mCINAP*^–/–^ mice (Figure 6G and H). Moreover, skeletal muscle-specific depletion of mCINAP decreased the lifespan of mice (Figure 6I). Taken together, these data indicate that mCINAP depletion aggravates senescence-associated phenotypes in mice and accelerates tissue senescence.

## Discussion

**Figure 7:**
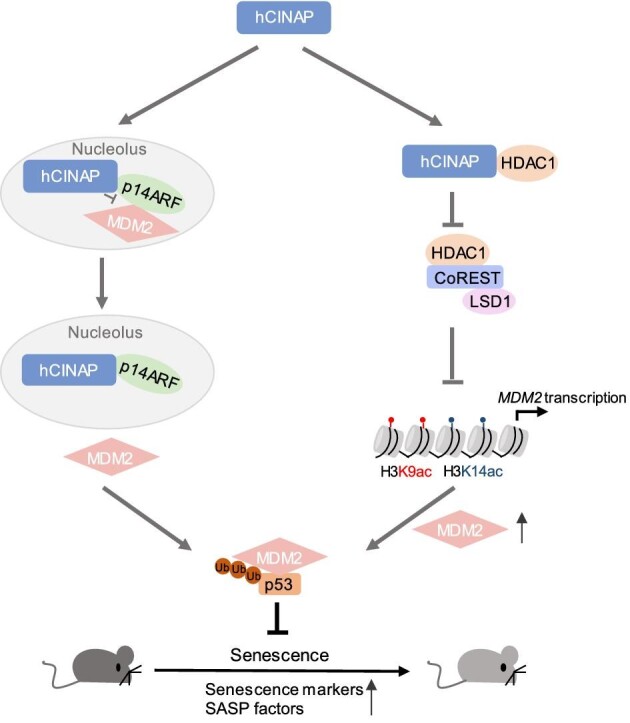
hCINAP delays aging through multiple mechanisms. On the one hand, hCINAP binds to p14ARF and promotes the release of MDM2 from the nucleolus into the nucleoplasm by inhibiting the p14ARF–MDM2 interaction. On the other hand, hCINAP interacts with HDAC1 and blocks the formation of the HDAC1/CoREST complex, which inhibits the deacetylation of H3K9ac in the *MDM2* promoter and results in an upregulation of *MDM2* transcription. Ultimately, the elevated levels of MDM2 promote the ubiquitination of p53 and decrease the stability of p53 to alleviate cellular senescence. Therefore, mCINAP deficiency in skeletal muscle/liver tissues increases the levels of senescence markers and SASP factors, accelerating tissue senescence in radiation-challenged mice.

Here, we demonstrate hCINAP as a negative regulator of aging and elucidate the mechanisms underlying the alleviation of senescence by hCINAP in primary cells and *in vivo* mouse models of aging. We propose a sequential working model of senescence, in which hCINAP levels are substantially decreased in senescent cells, and the subsequent initiation and progression of senescence are dependent on the hCINAP-modulated MDM2–p53 pathway. hCINAP interacts with p14ARF and promotes the release of MDM2 from the nucleolus into the nucleoplasm by attenuating the p14ARF–MDM2 interaction. In addition, hCINAP leads to the upregulation of *MDM2* transcription by blocking HDAC1/CoREST complex assembly and subsequently inhibiting the deacetylation of H3K9ac in the *MDM2* promoter. Ultimately, the increased levels of MDM2 promote ubiquitination of p53, which decreases its stability and delays cellular senescence. mCINAP deficiency in the skeletal muscle/liver results in accelerated tissue senescence, decreased grip strength, and reduced motor skills in radiation-challenged mice (Figure 7).

Recent studies demonstrate that hCINAP plays important roles in innate immunity, ribosome synthesis, and tumorigenesis (Bai et al., 2016; Ji et al., 2017; Xu et al., 2019). Our results indicate that hCINAP functions as a key regulator of aging. Both mRNA and protein expression levels of hCINAP decrease during primary cell aging, and knockdown of hCINAP facilitates the cellular aging process, suggesting that hCINAP expression may be an indicator of cellular senescence. Moreover, radiation-challenged mice with mCINAP deficiency in the skeletal muscle/liver display a more severe state of tissue aging than radiation-challenged control mice. These findings suggest that hCINAP also functions in *in vivo* senescence; however, whether mCINAP knockout-induced tissue aging accelerates aging in mice by secreting a large number of SASP factors remains to be studied.

p14ARF is an alternative reading-frame product of the *INK4/ARF* locus (Gil and Peters, 2006) and functions as a tumor suppressor by stabilizing and activating p53, which leads to cellular senescence and prevents tumor cell growth. Recent studies show that genetic variants in the *INK4/ARF* locus increase the risk of developing aging-related diseases (Liu et al., 2009). Activation of the INK4–ARF pathway triggers protective mechanisms against tumor-induced stress, which can also achieve anti-aging activity by reducing the damage in age-related phenotypes. Moreover, it has been reported that the p14ARF–MDM2–p53 axis is closely associated with aging (Varela et al., 2005; Seo et al., 2020). These findings indicate that aging and other pathological states are modulated by the regulatory response pattern of p53 downstream of p14ARF signaling. Here, we demonstrate that hCINAP promotes MDM2-dependent p53 degradation to alleviate cellular senescence by inhibiting the interaction between p14ARF and MDM2, establishing a potential link between hCINAP and the p14ARF–MDM2–p53 pathway. Additionally, previous studies have reported that phosphorylation of MDM2 plays an important role in the regulation of the MDM2–p53 pathway, i.e. phosphorylation of different MDM2 residues has diverse consequences on p53-mediated cellular proliferation, tumorigenesis, and senescence (Gannon et al., 2012; Chibaya et al., 2021). Moreover, phosphorylation of the N-terminus of p53 disrupts the MDM2–p53 interaction and accelerates the aging phenotypes (Craig et al., 1999; Maier et al., 2004). hCINAP is an adenylate kinase that promotes LDHA activity by enhancing phosphorylation at Y^10^ (Ji et al., 2017); therefore, whether hCINAP regulates MDM2–p53 signaling and p53-mediated senescence by phosphorylating residues in MDM2 or p53 to modulate the p14ARF–MDM2 interaction should be studied in the future.

Recent evidence suggests that HDAC1 plays important roles in cellular senescence, myelination, and neurogenesis by regulating several signaling pathways (Willis-Martinez et al., 2010). HDAC1 represses transcription by associating with certain DNA-binding proteins involved in complexes such as Sin3, NuRD, and CoREST (Glozak et al., 2005). In the present study, we demonstrate that hCINAP inhibits the deacetylation of H3K9ac in the *MDM2* promoter by blocking HDAC1/CoREST complex assembly and, in turn, upregulating *MDM2* transcriptional levels (Figure 5). Further investigation into whether the transcription of other genes involved in cellular senescence is regulated by hCINAP through disrupting HDAC1/CoREST complex assembly will deepen our understanding of the mechanisms underlying hCINAP function in aging.

It is worth mentioning that MDM2 increased in the nucleoplasm but decreased in the nucleolus in cells overexpressing hCINAP (Figure 4A and B), indicating that hCINAP attenuates the p14ARF–MDM2 interaction in the nucleolus. The HDAC1/CoREST complex is known to repress transcription by deacetylating and demethylating histone in the nucleoplasm, and the nucleoplasm-localized hCINAP interferes with the assembly of the HDAC1/CoREST complex. Collectively, different populations of hCINAP, localized in the nucleolus and nucleoplasm, interfere with the p14ARF–MDM2 interaction and HDAC1/CoREST complex assembly, respectively.

Overall, our data show that hCINAP alleviates senescence by multiple mechanisms. hCINAP promotes MDM2-mediated p53 ubiquitination and degradation not only by inhibiting the p14ARF–MDM2 interaction but also via the upregulation of *MDM2* transcription by blocking the HDAC1/CoREST complex-mediated deacetylation of H3K9ac. Our findings provide mechanistic insight into the functions of hCINAP in cellular and *in vivo* senescence.

## Materials and methods

### Cell culture and the generation of primary cells

HEK293T and HeLa cells were cultured in Dulbecco's modified Eagle's medium (DMEM; Gibco) supplemented with 10% fetal bovine serum (FBS; Gibco). Primary MEFs were isolated from 13.5-day-old embryos of C57BL/6 mice. IMR90 and MEF primary cells were cultured in DMEM supplemented with 10% FBS and 1% MEM nonessential amino acid solution (Solarbio). HEK293T cells were transfected using polyethylenimine (Polyscience) according to the manufacturer's protocol.

To create replicative senescence, confluent MEFs and IMR90 cells were each transferred into two dishes and cultured to confluence in order to produce one population doubling (PD). The assay for the number of PDs was performed according to a previously published method (Lyu et al., 2018).

### Treatment of IMR90 primary cells

IMR90 primary cells were grown to 70%–80% confluence and treated with DOX (2 μg/ml) for 24 h or irradiated (5 Gy). Fresh medium was added, and the cells were cultured for further 3–5 days to establish stress-induced senescence. Subsequently, cell lysates were subjected to immunoblotting.

### SA-β-gal staining

A total of 5 × 10^5^ MEFs or IMR90 cells were seeded on 6-well plates and grown to 50%–60% confluence. Cells were washed twice with 1 ml PBS/well, after which 1.5 ml 1× Fixation Buffer/well was added and allowed to incubate for 6–7 min at room temperature. During the fixation process, the staining mixture was prepared according to the manufacturer's instructions (Sigma Aldrich, CS0030). Cells were washed three times with 1 ml PBS/well and incubated overnight with the staining mixture at 37°C without CO_2_. The 6-well plates were sealed with Parafilm™ to prevent drying out. Cells were observed under a microscope at 10 × 10 magnification (Leica DMI 6000B, Leica). SA-β-gal signals were quantitated using the ImageJ software (NIH).

### Duolink® PLA

The *in situ* interaction between hCINAP and p14ARF was assessed using the Duolink® In Situ PLA® kit according to the manufacturer's instructions (Sigma-Aldrich, DUO92101). IMR90 cells were fixed in 4% paraformaldehyde for 10 min at room temperature and subsequently blocked with 1× blocking solution. Cells were incubated overnight at 4°C with primary antibodies against hCINAP and p14ARF, followed by incubation with PLA probes at 37°C for 1 h. Cells were washed three times, incubated with ligation-ligase solution at 37°C for 30 min, and subsequently incubated with amplification-polymerase solution at 37°C in the dark for a further 100 min. Cells were stained with mounting medium containing 4′,6-diamidino-2-phenylindole (DAPI). Fluorescence images were obtained under a confocal laser-scanning microscope (Zeiss LSM 710) using a 63× oil objective lens.

### ChIP–qPCR

Liver tissues used for the ChIP assay were homogenized by centrifugation (HODER N9548R), fixed in 1% formaldehyde, and quenched by the addition of 0.125 M glycine to the media. Samples were centrifuged at 2500 rpm for 5 min at 4°C and gently lysed in 0.5 ml NP40 lysis buffer for 5 min on ice. The lysate was overlaid on 1.25 ml sucrose and centrifuged at 12000 rpm for 10 min at 4°C to isolate the nuclei. The pellet was then sequentially resuspended in glycerol buffer and nuclei lysis buffer prior to resuspension in 1 ml sonication buffer containing protease inhibitor cocktail. Released chromatin was disrupted using a DNA-shearing sonicator (Qsonica Q800R3). Samples were incubated with specific antibodies at 4°C overnight with rotation, and the chromatin–antibody complexes were captured using Protein G Dynabeads™. After the phenol chloroform extraction step, ChIP DNA was used for qPCR. The primers used for ChIP–qPCR are listed in Supplementary Table S1.

### Animal irradiation

Mice were housed in a specific pathogen-free barrier facility and handled according to the ‘Principles for the Utilization and Care of Vertebrate Animals’ and the ‘Guide for the Care and Use of Laboratory Animals’. Animal studies were approved by the Institutional Animal Care and Use Committee (IACUC) of the Center for Experimental Animal Research (China) and Peking University Laboratory Animal Center (IACUC No. LSCZhengX-2-1). m*CINAP*^+/+^ and skeletal muscle/liver-specific *mCINAP*^–/–^ C57BL/6J mice (males) at 3 months of age were exposed to a sublethal dose (5 Gy) of irradiation to induce *in vivo* senescence. At 1, 2, 4, 6, and 9 months after irradiation, mice were sacrificed and various tissues were collected for immunoblotting, RNA extraction, immunohistology, and SA-β-gal staining. Mice in the control and experimental groups were age-matched males.

### Measurement of grip strength

Grip strength was measured using the Ugo Basile grip strength test device. Mice were grabbed by the tail and allowed to grasp the metal grid with their two front paws. The force strength shown on the device by pulling back in the horizontal plane until the mouse released the grid was recorded.

### Rotarod

Mice were trained to run on the rotarod instrument (ENV-574 M, MED Associates) in three daily trials at a fixed speed (4 rpm) for 300 sec. The training lasted for 3 days. During this training period, mice that fell were placed back on the rod until the end of 300 sec. In subsequent experiments, mice were tested in a session of three consecutive trials on an accelerating rotarod (4–40 rpm for >5 min). The interval between trials is 15 min. Latency to fall was recorded for each animal and averaged over the three trials. The test lasted for 3 days. The same cohort of radiation-challenged control and skeletal muscle-specific *mCINAP^–/–^* mice were used for this experiment.

### Statistical analysis

Statistical analysis was performed using Student's *t*-test unless otherwise specified. Data are expressed as mean ± standard error of the mean (SEM). *P < 0.05, **P < 0.01, ***P < 0.001, ****P < 0.0001.

## Supplementary Material

mjad015_Supplemental_File

## Notes

**Author contributions:** X.H. and X.Z. designed this study. X.H., Y.Z., M.W., and R.Z. performed the experiments. X.H. analyzed the data and wrote the manuscript. X.Z. supervised this study and wrote the manuscript.
