# Supplementary material for: hCINAP alleviates senescence by regulating MDM2 via p14ARF and the HDAC1/CoREST complex
Source: J Mol Cell Biol. 2023 Mar 6;15(2):mjad015. doi: 10.1093/jmcb/mjad015 (PMC10476552; doi:10.1093/jmcb/mjad015)
Supplement: mjad015_Supplemental_File [file mjad015_supplemental_file.pdf]

Supplementary information

Antibodies and Reagents

Mouse monoclonal anti-HA (H9658) and anti-Flag (F3165) antibodies were purchased from Sigma-Aldrich. Mouse monoclonal anti-Myc (M047-3) and anti-His (D291-3) were bought from MBL. Rabbit polyclonal anti-β-actin (AC026), anti-CDKN1A (A1483), anti-CDKN2A (A0262), and anti-TP53 (A5761) were procured from ABclonal Technology. Mouse monoclonal anti-MDM2 (SMP14) and anti-p14ARF (DCS-240) were obtained from Santa Cruz Biotechnology. Rabbit monoclonal anti-Histone H3 (acetyl K9) (ab32129) was purchased from Abcam. Rabbit polyclonal anti-AK6 (G35) was bought from Bioworld Technology. Mouse monoclonal anti-p53 (60283-2-Ig), rabbit polyclonal anti-Ki67 (27309-1-AP) and anti-NCL (10556-1-AP) were procured from Proteintech.

Bacto™ Peptone (211677) and Bacto™ Agar (214010) were obtained from BD (Becton Dickinson and Company). The senescent cell histochemical staining kit (CS0030) was purchased from Sigma-Aldrich. Ni^{+}-NTA was bought from Qiagen, and Protein G Sepharose™ 4B was procured from GE Healthcare. The transfection reagent PEI was obtained from Polyscience. CHX (C7698) and puromycin (P8833) were purchased from Sigma-Aldrich. MG132 (T2154) was bought from TargetMol. Polybrene (sc-134220) was procured from Santa Cruz Biotechnology.

Plasmids

hCINAP and MDM2 were cloned into the 3×Flag-pcDNA and 3×HA-pCMV vectors, respectively. P14ARF was cloned into the 3×Myc-pcDNA vector. His-tagged p14ARF was cloned into the pET-28a vector. GST-hCINAP was constructed by inserting the cDNA encoding hCINAP into the pGEX-4T-1 vector. All plasmids were confirmed by DNA sequencing.

Quantitative real-time PCR analysis

Total RNA was extracted using TRIzol™ reagent (Invitrogen) according to the manufacturer's instructions. RNA was reverse-transcribed to cDNA using random hexamer primers and the FastQuant RT kit (TIANGEN). cDNA was used as the template for RT-qPCR and amplified with the aid of a fast two-step amplification program, specific primers, and SYBR™ Green Master Mix (YEASEN), and measured

by qPCR (Lightcycler, Roche). Data were normalized to β-actin as the internal control. The primers used in this analysis are shown in Table S1.

His-ubiquitin pulldown assay

At 70–80% confluence, HEK293T cells were transfected with His-ubiquitin and other indicated plasmids. At 36 h post-transfection, cells were treated with 10 μM MG132 for 8 h and harvested in lysis buffer. His-ubiquitin pulldown was performed according to a previously described method (Li et al., 2014).

Co-immunoprecipitation

To detect the interaction between hCINAP and p14ARF, HEK293T cells were harvested and lysed in RIPA buffer at 4℃ for 2 h. Subsequently, the lysate was incubated with the indicated antibody or IgG at 4 ℃ for 4 h, after which 30 μL protein G beads was added and the lysate was shaken at 4 ℃ for 4 h. Finally, the beads were washed three times in RIPA buffer and denatured in 2× SDS loading buffer. The binding of hCINAP and p14ARF was examined by immunoblotting using the indicated antibodies.

Lentivirus infection

The lentiviral PLKO.1 vectors for knockdown of hCINAP and p14ARF were constructed in our laboratory (Yang *et al*., 2018). hCINAP knockdown was performed using lentiviruses, shhCINAP #1 (5'-CAGAGUAGUUGAUGAGUUA-3') and shhCINAP #2 (5'-GAGAGAAGGUGGAGUUAUU-3'), which were constructed in the PLKO.1 vector (Addgene). p14ARF was depleted using the shRNA shp14ARF #1 (5'-GCAGTAACCATGCCCGCATAG-3'). All PLKO.1 plasmids were packaged into the lentiviral system using the VSVG, PLP1, and PLP2 vectors. A scrambled shRNA lentivirus with no effect on hCINAP or p14ARF was constructed as a negative control using the following sequence: 5'-TTCTCCGAACGTGTCACGT-3' (GeneChem Co. Ltd). Cells were infected with the packaged viruses using Polybrene® (5 μg/mL). At 36–48 h post-infection, cells were cultured in medium containing puromycin (1 mg/mL) for a further 24–36 h to select stable clones. Subsequently, cells were plated onto fresh dishes. Lentiviruses overexpressing shRNA-resistant hCINAP were constructed using the pLVX-IRES-ZsGreen1 vector. Clones exhibiting stable knockdown or overexpression were identified by immunoblotting.

Protein purification

Recombinant GST-hCINAP fusion proteins were expressed in *E. coli* BL21 (DE3) cells and purified using Glutathione Sepharose® 4B (GE Healthcare). His-tagged p14ARF proteins were also expressed in *E. coli* BL21 (DE3) cells and purified using Ni(ii) Sepharose® (GE Healthcare). For the *in vitro* pulldown assay, the GST-hCINAP fusion protein was incubated with His-tagged p14ARF in modified PBS buffer (137 mM NaCl, 2.7 mM KCl, 10 mM Na_2HPO_4, 2 mM KH_2PO_4, 1% Triton X-100, 4 mM EDTA, 1 mM DTT, pH 7.4) at 4 ℃ for 2 h. The reaction system was washed four times with modified PBS buffer, and the interaction between hCINAP and p14ARF was examined by immunoblotting using the indicated antibodies.

Determination of the lifespan of *C. elegans*

C. elegans were maintained and grown on Nematode Growth Medium (NGM) agar plates at 20 ℃ using *E. coli* OP50 bacteria as a food source. HT115 bacteria were transformed with the L4440-ADLP plasmid; the L4440 plasmid was used as a negative control. Subsequently, HT115 bacteria were cultured in 3 mL LB liquid medium with shaking. After reaching an OD_{595} of 0.4, 1 mM IPTG was added to the medium and the bacteria were cultured for a further 4 h. The bacterial suspension was spread on the plate and cultured for 16–48 h. The pregnant worms were treated with lysate to release the eggs, which were then transferred to the NGM plates and cultured to L4 larvae at 20 ℃. The F1 generation was transferred to fresh plates and counted every other day. Fresh plates were prepared rapidly and FUDR was used to prevent reproduction. The lifespan was calculated using the GraphPad Prism Software.

Immunofluorescence microscopy

Cells were plated on coverslips (24 mm × 24 mm) in 6-well plates. At 60–70% confluence, the culture medium was removed and the coverslips were washed three times with ice-cold PBS. Cells were fixed in 4% paraformaldehyde (P0099, Beyotime) for 20 min at room temperature, washed three times with PBS, and transferred to a damp dish. Cells were blocked in 1% BSA at room temperature for 1 h and incubated overnight at 4 ℃ with primary antibodies (anti-hCINAP, anti-MDM2, and anti-NCL) diluted in 1% BSA. The next day, cells were washed three times with PBS and incubated at room temperature for 1 h with FITC/TRITC-conjugated secondary

antibodies diluted in 1% BSA. Subsequently, cells were washed three times with PBS and incubated with DAPI at room temperature for 10 min. Images were captured using a two-photon confocal laser-scanning microscope (Zeiss LSM 710).

Immunohistochemistry

Skeletal muscle and liver tissues were isolated from skeletal muscle/liver-specific *mCINAP*$^{-/-}$ mice. Slides were stained with mouse anti-p53 (1:100), or rabbit anti-IL-1α (1:100) at 4 ℃ overnight, followed by incubation with the secondary antibody at room temperature for 1 h. Reaction products were visualized using 3, 3'-diaminobenzidine. The negative control sample was treated identically, without the primary antibody. Immunostaining was evaluated blindly by pathologists from Peking University Health Science Center based on the histochemical score. The intensities of p53 and IL-1α staining were classified as follows: 0, no staining; 1, weak reactivity; 2, moderate reactivity; 3, strong reactivity; 4, very strong reactivity. Images were obtained using a Leica DM IRE2 microscope.

Table S1
Primers for Real-time RT-qPCR

Name	Primer sequence (S)	Primer sequence (AS)
MDM2 F1	CAGAAATCCACCTGCCTCTG	TGACCCCTGCAGTAGAGACG
MDM2 F2	AAGGTCTATGCCACCATGCC	CTGGGTCCACCTTTTAAGGT
MDM2 F3	ACGGCTGTTGCTCTGTACCT	TAGGGACATTCCATTCCCGG
MDM2 F4	GGACCCAATAGATTAACATAGAGGTC	GAATAGCTCCATGGTCAAGAGTG
MDM2 F5	CACTTTCCACCCCGCTCTCC	GCCGGAAGCCACGGGCCATC
hCINAP	GGTGGAGTTATTGTTGATTAC	CCTTGTAGGATGCTGTGGC
Human IL-1α	AGATGCCTGAGATACCCAAAACC	CCAAGCACACCCAGTAGTCT
Human CXCL-1	AGGGAATTCACCCCAAGAAC	TGGATTTGTCACTGTTCAGCA
Human IL-6	TGACCCAACCACAAATGC	CTGGCTCTGAAACAAAGGAT
Human IL-8	TGTGGGTCTGTTGTAGGG	GTGAGGTAAGATGGTGGC
Human β-actin	GTGGACATCCGCAAAGAC	AAAGGGTGTAACGCAACTAA
Mouse p16	CGCAGGTTCTTGGTCACTGT	TGTTCACGAAAGCCAGAGCG
Mouse p21	CCTGGTGATGTCCGACCTG	CCATGAGCGCATCGCAATC
Mouse IL-6	GGAAATCGTGGAAATGAG	GCTTAGGCATAACGCACT
Mouse IL-8	ATGGCTGGGATTCACCTC	TCGCACAACACCCTTCTA
Mouse β-actin	CACTGTGCCCATCTACGA	CAGGATTCCATACCCAAG

S, sense; AS, antisense

Figure legends

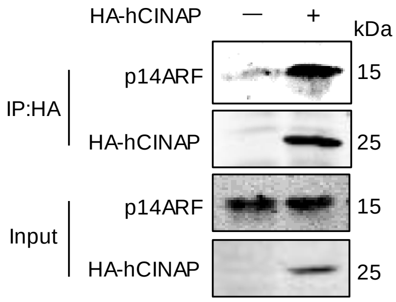

Figure S1. hCINAP interacts with p14ARF. HEK293T cells were transfected with HA-hCINAP or empty vector, and immunoprecipitation was performed using an anti-HA antibody to examine the interaction between HA-hCINAP and endogenous p14ARF.

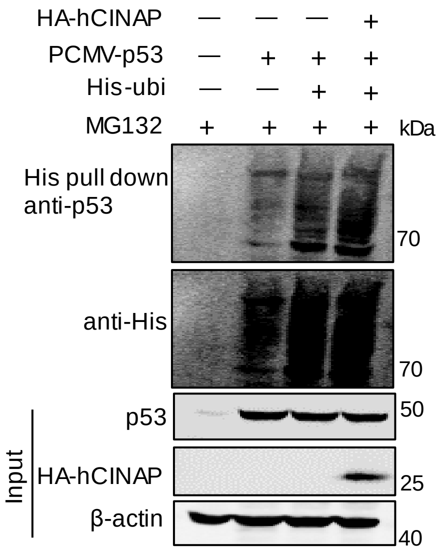

Figure S2. hCINAP promotes the ubiquitination of p53. HEK293T cells transfected with the indicated plasmids were subjected to His-ubiquitin pulldown.

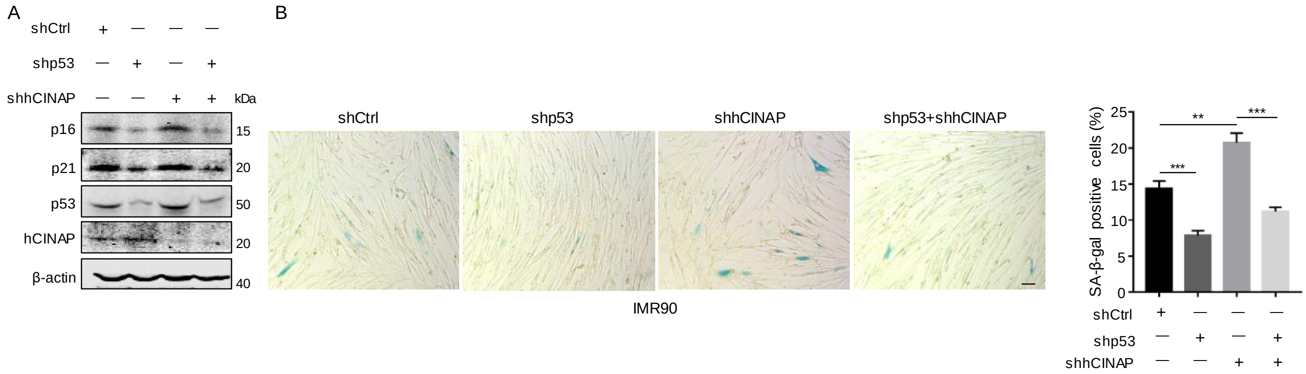

Figure S3. hCINAP-mediated IMR90 cell senescence is dependent on p53. (**A**) The protein levels of p21 and p16 in IMR90 cells as indicated by immunoblotting using the indicated antibodies. (**B**) SA-β-gal staining of control (shCtrl) and p53 and/or hCINAP-knockdown (shp53 and shhCINAP) IMR90 cells. Scale bar, 50 μm.

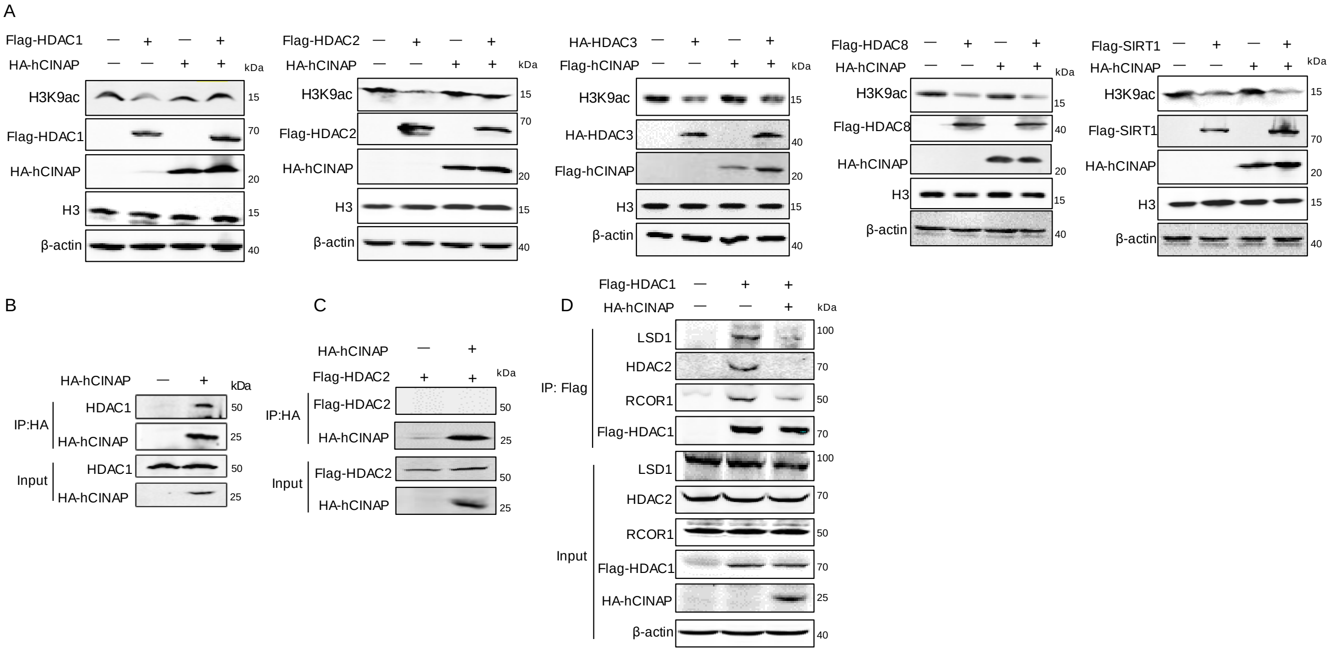

Figure S4. hCINAP inhibits the deacetylation of H3K9ac by interacting with HDAC1. (A) HEK293T cells transfected with the indicated plasmids were subjected to immunoblotting using the indicated antibodies to examine the effect of hCINAP on H3K9ac expression with or without HDAC1/HDAC2/HDAC3/HDAC8/SIRT1. (B) HEK293T cells transfected with the indicated plasmids was used to examine the interaction between HA-hCINAP and endogenous HDAC1 by immunoprecipitation using an anti-HA antibody. (C) HEK293T cells were transfected with the indicated plasmids, and immunoprecipitation was performed to examine the interaction between HA-hCINAP and Flag-HDAC2 using an anti-HA antibody. (D) Effect of hCINAP on the interaction between HDAC1 and the three major components (LSD1, HDAC2, and RCOR1) of the CoREST complex was examined by co-IP in HEK293T cells transfected with the indicated plasmids.

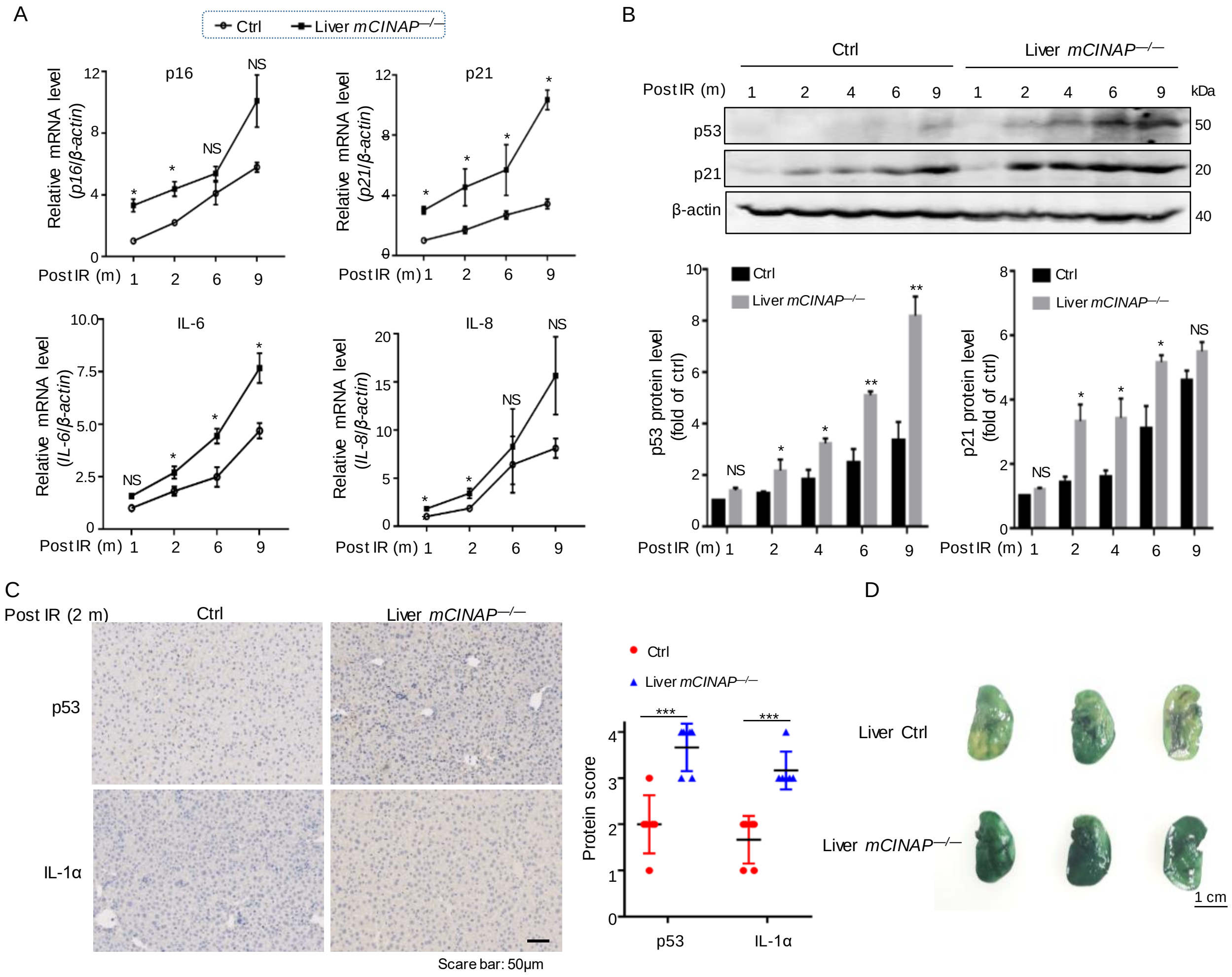

Figure S5. mCINAP depletion aggravates senescence-associated phenotypes in liver-specific *mCINAP*$^{-/-}$ mice. (**A**) Quantitative measurement of the mRNA expression of senescence markers (p16 and p21) and key SASP factors (IL-6 and IL-8) in the liver from control and liver-specific *mCINAP*-knockout mice. (**B**) Immunoblotting of liver extracts showing the protein expression levels of senescence markers (p16 and p21). (**C**) Immunohistochemistry of p53 and IL-1α expression (Scale bar, 50 μm) in the liver from control and liver-specific *mCINAP*-knockout mice. Statistical results were analyzed using the ImageJ software. (**D**) Representative images of SA-β-Gal staining in liver tissues from control and liver-specific *mCINAP*-knockout male mice (n = 3). Scale bar, 1 cm. Data in **A**, **B**, and **C** are expressed as the mean ± SEM (n = 3). Statistical analysis was performed using a Student's *t*-test. *$p < 0.1$, **$p < 0.01$, ***$p < 0.001$, NS, not significant.

References

Li, T., Guan, J., Huang, Z., et al. (2014). RNF168-mediated H2A neddylation antagonizes ubiquitylation of H2A and regulates DNA damage repair. J Cell Sci *127*, 2238-2248.

Yang, C., Zang, W., Tang, Z., et al. (2018). A20/TNFAIP3 Regulates the DNA Damage Response and Mediates Tumor Cell Resistance to DNA-Damaging Therapy. Cancer Res *78*, 1069-1082.
